# Genetically Modified (GM) Crop Use 1996–2020: Impacts on Carbon Emissions

**DOI:** 10.1080/21645698.2022.2118495

**Published:** 2022-10-11

**Authors:** Graham Brookes

**Affiliations:** PG Economics, Dorchester, UK

**Keywords:** Fuel use, herbicide, insecticide, list: GM crop, reduced and no tillage, soil carbon sequestration, weed resistance

## Abstract

This paper assesses how the use of genetically modified seed (GM) crop seed technology has impacted on greenhouse gas emissions at a global level. The main technologies of relevance are crops modified to be tolerant to specific herbicides so as to facilitate improved weed control and crops resistant to a range of crop insect pests that otherwise damage crops or typically require the application of insecticides to control them. Over the 24 year period examined to 2020, the widespread use of GM insect resistant and herbicide tolerant seed technology has led to important cuts in on-farm fuel use and facilitated farmers moving from plow-based systems to reduced and no tillage systems that they have continued to operate for a number of years. This has led to a significant reduction in the release of greenhouse gas emissions from the GM cropping area, which in 2020 was equal to a saving of 23,631 million kg of carbon dioxide, equivalent to taking 15.6 million cars off the road for a year (equal to 49% of registered cars in the UK).

## Introduction

Since 1996 when the first significant area of GM crops were planted (1.66 million hectares), there has been a major increase in plantings so that in 2020, the global area planted to crop varieties that contain GM traits was 185.6 million hectares (ha). These seed varieties were mostly found in the four crops of soybeans, maize/corn, cotton and canola, with just under 50% of the 2020 global area of these four crops having used GM-traited seed.

This paper discusses changes in farming practices arising from the growing of GM crops and how this has impacted on global Greenhouse Gas (GHG) emissions: It is widely accepted that increases in atmospheric levels of greenhouse gases such as carbon dioxide, methane and nitrous oxide are detrimental to the global environment (see, for example, Intergovernmental Panel on Climate Change 2006.^1^) Therefore, where the growing of GM crops has resulted in a reduction in the level of greenhouse gas emissions from agriculture, this represents a positive environmental development for the world.

The three main GHGs of relevance to agriculture are carbon dioxide (CO2), nitrous oxide (N2O) and methane (CH4). The scope for GM crops contributing to lowering levels of GHG comes from three principal sources:

- Reduced fuel use from fewer herbicide or insecticide applications;
- The use of ‘no-till’ and ‘reduced-till’ farming systems collectively referred to as conservation tillage (see below for definitions) has increased significantly with the adoption of genetically modified herbicide-tolerant (GM HT crops). The GM HT technology has improved farmers’ ability to control weeds, reducing the need to rely on soil cultivation and seed-bed preparation as means to getting good levels of weed control. The advantages of conservation tillage include:
  - Lower fuel costs (less soil preparation; plowing, harrowing, rolling etc.);
  - Reduced labor requirements associated with soil preparation;

- Enhanced soil quality and reduced levels of soil erosion, resulting in more carbon remaining in soil, which leads to lower GHG emissions;
- Improved levels of soil moisture conserving;
- Reduced soil temperature fluctuations from the insulating properties of crop residues. This has a positive impact on both the physical, chemical and microbiological properties of soil (Mathew et al. 2012^2^); and

- Additional carbon dioxide can be assimilated where the GM technology leads to the intensification of crop production resulting in higher crop yields, additional cropping and the use of cover crops. As indicated in a number of meta-analyses (e.g., Brookes and Barfoot, 2020a,^3^ Klumper and Qaim, 2014,^4^ Finger et al., 2011,^5^) the adoption of GM technology has resulted in additional production from a combination of higher yields and facilitation of second cropping of soybeans after a wheat crop in South America.

As estimating the possible GHG emissions savings associated with this additional production is difficult due to the complex array of variables that impact on this, which vary by location, no estimates are provided in this paper. This paper therefore examines only the scope for reductions in GHGs in terms of the amount of carbon dioxide removed from the atmosphere by reduced consumption of fuel and additional storing and sequestration of carbon in the soil with NT/RT tillage practices.

In this paper, soil tillage systems have been differentiated into three categories depending upon their impact on soil disturbance:

- Conventional tillage (CT): conventionally tilled prior to planting the next crop (residue cover 0–15%) e.g., inversion tillage using a plow followed by multiple cultivation trips;
- Reduced tillage (RT): full width tillage that disturbs the entire soil surface prior to planting the next crop, tillage tools such as chisel plows, field cultivators, rotary harrows are used and weeds are controlled by cultivation and herbicides. With RT methods of mulch-till and ridge till, crop residue remains on the surface (this corresponds to a residue cover of 16–30% for all crops other than maize, for which there is a reduced tillage category with a higher crop residue cover of 31–50%); and
- No-till (NT): the least intensive form of tillage where a minimal amount of soil disturbance is made to ensure a good crop stand and yield. NT methods include zero-till, slot till, direct seeding and strip-till. The soil is not tilled prior to planting the next crop and substantial crop residue remains on the surface (this corresponds to a residue cover of >30% for all crops other than maize, for which the residue cover is >50%).

The study integrates data for 2019 and 2020 into the context of earlier developments and updates the findings of earlier analysis presented by Brookes and Barfoot, 2020b.^6^

## Methodology

The assessment of how the planting of GM crops has impacted on greenhouse gas emissions is based on a literature review relating to fuel use and tillage systems and how these have changed due to the use of GM crop technology. Reductions in the level of GHG emissions associated with an increase in the area of NT and RT tillage facilitated by the planting of GM crops are acknowledged in a wide body of literature (Conservation Tillage and Plant Biotechnology (CTIC), 2002,^7^ American Soybean Association Conservation Tillage Study, 2001,^8^ Reicosky, 1995,^9^ Robertson et al., 2000,^10^ Johnson et al., 2005,^11^ Derpsch et al., 2010,^12^ Eagle et al., 2012,^13^ Olson et al., 2013,^14^ Perry et al., 2016).^15^

### Fuel Savings

GM crops contribute to a reduction in fuel use from less frequent herbicide or insecticide applications and a reduction in the energy use in soil cultivation. The move away from a plow-based, to a RT/NT production system has led to a reduction in fuel use. The fuel savings used in this paper are drawn from a review of literature, in particular the USDA’s Conservation Effects Assessment Project (CEAP: 2016,^16^) CTIC 2002,^8^ USDA Energy Estimator for Tillage Model 2013^17^ and the online USDA Comet-VR model 2013.^18^ In this analysis, it is assumed that the adoption of NT farming systems in soybean production reduces cultivation and seedbed preparation fuel usage by 27.12 liters/ha compared with traditional conventional tillage and in the case of RT (mulch till) cultivation by 10.39 liters/ha. In the case of maize, NT results in a saving of 24.41 liters/ha and 7.52 liters/ha in the case of RT compared with conventional intensive tillage. These estimates are in line with the USDA Energy Estimator for soybeans and maize. In terms of GHG, each liter of tractor diesel consumed contributes an estimated 2.67 kg of carbon dioxide into the atmosphere (US EPA Greenhouse Gas Equivalencies Calculator.^19^) The adoption of NT and RT systems in respect of fuel use therefore results in reductions of carbon dioxide emissions of 72.41 kg/ha and 27.74 kg/ha, respectively, for soybeans and 65.17 kg/ha and 20.08 kg/ha for maize.

The fuel saving associated with changes in the number of herbicide and insecticide applications depends upon the application method. For example, in the USA, a typical method of application is with a 90-foot boom sprayer which consumes approximately 0.84 liters/ha (Lazarus, 2019).^20^ Our analysis uses this value and based on this, in terms of GHG, each liter of tractor diesel consumed contributes an estimated 2.67 kg of carbon dioxide into the atmosphere (so one less application reduces carbon dioxide emissions by 2.24 kg/ha). As many farmers apply insecticides via sprayers pulled by tractors, which use higher levels of fuel than self-propelled boom sprayers, the estimates made for reductions in carbon emissions, which are based on self-propelled boom application, therefore, probably understate the full carbon dioxide savings. Readers should note that the analysis also excludes consideration of any carbon emission savings associated with reduced use of insecticides on crops like cotton in developing countries because most of these applications are made by hand and do not use any fuel during application.

### Soil Carbon Sequestration, Tillage and GM HT Crops

The use of RT/NT farming systems increases the amount of organic carbon in the form of crop residue that is stored or sequestered in the soil and therefore reduces carbon dioxide emissions to the environment (Intergovernmental Panel on Climate Change, 2006,^1^ Robertson et al., 2000,^10^ Johnson et al., 2005,^11^ Calegari et al., 2008,^21^ Baker et al., 2007,^22^ Angers and Eriksen-Hamel, 2008,^23^ Blanco-Canqui and Lal, 2008,^24^ Lal, 2004,^25^ Lal, 2005,^26^ Lal, 2010,^27^ Bernaccchi et al., 2005,^28^ Michigan State University, 2016,^29^) Buragiene *et al*. 2019,^30^ Mangalassery *et al*. 2014,^31^ Nicoloso & Rice 2019^32^ and Haruna & Nkongolo 2019.^33^

Complex models are available to estimate the level of carbon sequestered depending upon historic, present and future cropping systems. For example, the USDA’s COMET-Planner^18^ applies emission reduction coefficients for changes in tillage practice from CT to NT and RT based on a meta-analysis of the relevant literature. Its estimates are based on three key soybean and maize production states and assumes the adoption of NT from CT in all states, a clay loam soil with average fertilizer usage, a non-irrigated maize-soybean rotation in Minnesota and Illinois and a soybean-maize-winter-wheat rotation in South Dakota. The level of carbon sequestered estimated to be stored is higher with NT by 117.5, 114.4 and 112.9 kg carbon/ha/year, respectively, compared to the CT system for each of the three states for the projected period 2013–2023.

Analysis using the Michigan State University – US Cropland Greenhouse Gas Calculator (http://surf.kbs.msu.edu/)^29^ for maize-soybean rotations in the same locations over a ten-year projected period estimated that NT sequesters an additional 123 kg carbon/ha/year compared to RT and 175 kg carbon/ha/year compared to CT. Analysis of individual crops using the Michigan State University – US Cropland Greenhouse Gas indicates that NT maize is a net carbon sink of 244 kg carbon/ha/year, whereas, NT soybean is a marginal net source of carbon of 43 kg carbon/ha/year. The difference between maize NT and CT is 247 kg carbon/ha/year and for soybeans 103 kg carbon/ha/year.

Differences in carbon soil sequestration rates between maize and soybeans can be partially

explained by the greater plant matter residue contribution of the maize crop in the soybean-maize rotation. Alvarez & Steinbach 2012^34^ estimated that maize contributes 7,178 Mg/ha/year of dry matter as crop residue compared to soybeans which contribute only 3,373 Mg/ha/year. Soybean roots have less mass and length than maize roots which may also influence different rates of soil carbon sequestration.

Overall, the literature highlights the difficulty in estimating the contribution NT/RT systems to soil carbon sequestration levels. If a specific crop area is in continuous NT crop rotation, the full soil carbon sequestration (storage) benefits described in the literature can be realized. However, if the NT crop area is returned to a conventional tillage system, a proportion of the soil organic carbon gain will be lost. The temporary nature of this form of carbon storage only becomes permanent when farmers adopt a continuous NT system, which, as indicated earlier, is highly reliant on having an effective (typically herbicide-based) weed control system.

GM HT (tolerance to glyphosate) based production systems have provided this highly effective weed control system and the importance of this technology in the adoption of NT/RT systems was first highlighted by the American Soybean Association 2001.^8^ This study found that the availability of GM HT soybeans facilitated and encouraged farmers to implement reduced tillage practices; a majority of growers surveyed indicated that GM HT soybean technology had been the factor of greatest influence in their adoption of reduced tillage practices. Fernandez-Cornejo et al. 2012^35^ also concluded over an eleven-year period (1996–2006) that GM HT soybean adoption had led to a significant increase in the adoption of conservation tillage (RT/NT) with a one percent increase in GM HT soybean adoption leading to a 0.21% increase in conservation tillage. Similarly, Finger et al. (200 9^36^: based on a survey of Argentine soybean growers) identified that the combination of herbicide tolerance and NT were the key drivers to adoption of GM HT soybeans, facilitating easier crop management and reducing herbicide costs.

Although GM HT technology has played an important role in facilitating farmers adopting and, more importantly staying in NT/RT production systems, the increasing emergence of weeds resistant to glyphosate (the main herbicide used for ‘over the crop’ weed control in GM HT crops) has reduced the effectiveness of weed control systems solely based on herbicide use for some farmers and resulted in some reversion to CT production systems in order to improve their overall levels of weed control. This has likely reduced the year on year absolute levels of carbon sequestration facilitated by GM HT crops relative to several years ago in some countries (see, for example, Lu et al. 2022,^37^ Van Deynze et al. 2021.^38^) The year on year estimates presented in this paper (see appendix 1 for details) take this factor of influence into account by using the latest available data on the adoption of NT, RT and CT production systems although all subsequent estimates for possible cumulative soil carbon sequestration benefits are caveated to recognize this factor of influence.

Drawing on the literature and models referred to above, the analysis presented in the following sub-sections assumes the following:

*US*: The soil carbon sequestered by tillage system for corn in continuous rotation with soybeans is assumed to be a net sink of 250 kg of carbon/ha/year based on:

- NT systems store 251 kg of carbon/ha/year;
- RT systems store 75 kg of carbon/ha/year;
- CT systems store 1 kg of carbon/ha/year.

The soil carbon sequestered by tillage system for soybeans in a continuous rotation with corn is assumed to be a net saving of 100 kg of carbon/ha/year (all soybean systems release some carbon but relative to CT systems, RT and NT release less) based on:

- NT systems release 45 kg of carbon/ha/year;
- RT systems release 115 kg of carbon/ha/year;
- CT systems release 145 kg of carbon/ha/year.

*Argentina and Brazil*: soil carbon retention is 175 kg carbon/ha/year for NT soybean cropping and CT systems release 25 kg carbon/ha/year (a difference of 200 kg carbon/ha/year). This is a conservative estimate based on Alvarez et al., 2014.^39^

Overall, the GHG emission savings derived from reductions in fuel use for crop spraying have been applied only to the area of GM IR crops worldwide (but excluding countries where conventional spraying has traditionally been by hand, such as in India and China) and the savings associated with reductions in fuel from less soil cultivation plus soil carbon storage have been limited to NT/RT areas in North and South America that have utilized GM HT technology. Lastly, some RT/NT areas have also been excluded where the consensus view is that GM HT technology has not been the primary reason for use of these NT/RT systems (e.g., parts of Brazil).

## Results and Discussion

### Reduced Fuel Use

The fuel savings arising from making fewer insecticide applications with the use of GM IR crop technology in maize, cotton and soybeans (in countries where farmers previously used mechanical forms of application) and the switch from conventional tillage to reduced tillage or no tillage systems facilitated by GM HT crops have delivered permanent savings in carbon dioxide emissions. Over the period 1996 to 2020, the cumulative permanent reduction in fuel use has been about 39,147 million kg of carbon dioxide, arising from reduced fuel use of 14,662 million liters. In terms of car equivalents, this is equal to taking 25.9 million cars off the road for a year (Table 1).

**Table 1.** Carbon storage/sequestration from reduced fuel use with GM crops 1996–2020.

Crop/trait/country	Fuel saving (million liters)	Permanent carbon dioxide savings arising from reduced fuel use (million kg of carbon dioxide)	Permanent fuel savings: as average family car equivalents removed from the road for a year (‘000s)
**HT soybeans**			
Argentina	4,433	11,837	7,844
Brazil	2,749	7,341	4,865
Bolivia, Paraguay, Uruguay	899	2,401	1,591
US	1,687	4,503	2,984
Canada	255	681	451
**HT maize**			
US	2,257	6,027	3,994
Canada	121	323	214
**HT canola**			
Canada: GM HT canola	1,067	2,848	1,887
**IR maize**			
Brazil	369	984	652
US/Canada/Spain/South Africa	91	243	161
**IR cotton – global**	285	760	504
**IR soybeans – South America**	449	1,199	795
***Total***	***14,662***	***39,147***	***25,942***

The largest fuel use-related reductions in carbon dioxide emissions have come from the adoption of GM HT technology and how it has facilitated a switch to RT/NT production systems with their reduced soil cultivation practices. This accounted for 92% of the fuel and carbon dioxide savings in the period 1996–2020, within which GM HT soybeans accounted for the largest contribution (68% of the total savings). These savings have been greatest in South America.

In 2020, the fuel-related savings were 2,330 million kg of carbon dioxide, arising from reduced fuel use of 948 million liters. These savings are equivalent to taking 1.68 million cars off the road for one year.

Additional detail relating to the estimates for these carbon dioxide savings at the country and trait levels are presented in Appendix 1.

1. Assumption: an average family car in 2020 produces 123.4 grams of carbon dioxide per km. A car does an average of 12,231 km/year and therefore produces 1,509 kg of carbon dioxide/year
2. GM IR cotton. India, Pakistan, Myanmar and China excluded because insecticides assumed to be applied by hand, using back pack sprayers

### Additional Soil Carbon storage/sequestration

As indicated above, the widespread adoption and (more importantly) maintenance of RT/NT production systems in North and South America has been significantly facilitated by the availability of GM HT crop technology (especially in soybeans). As a result, as well as the tractor fuel use for tillage having been reduced, soil quality has been enhanced and levels of soil erosion cut. In turn, more carbon has remained stored in the soil leading to lower emissions of carbon dioxide.

Based on the areas of GM HT crops using RT/NT production systems in North and South America in 2020, we estimate that an extra 5,750 million kg of soil carbon was sequestered in 2020. This is equivalent to 21,101 million kg of carbon dioxide that has not been released into the global atmosphere. In terms of removing vehicles from the road, these savings are equivalent to taking 14 million cars off the road for one year (Table 2).

**Table 2.** Context of carbon sequestration impact 2020: car equivalents.

Crop/trait/country	Additional carbon stored in soil (million kg of carbon)	Potential additional soil carbon sequestration savings (million kg of carbon dioxide)	Soil carbon sequestration savings: as average family car equivalents removed from the road for a year (‘000s)
**HT soybeans**			
Argentina	1,832.5	6,725.2	4,445.8
Brazil	1,485.0	5,450.1	3,611.0
Bolivia, Paraguay, Uruguay	490.7	1,800.8	1,193.1
US	110.9	407.0	269.6
Canada	62.9	230.7	152.9
**HT maize**			
US	1,481.6	5,437.6	3,602.7
Canada	15.6	57.4	38.0
**HT canola**			
Canada: GM HT canola	270.4	992.4	657.5
**IR maize**			
Brazil	0	0	0
US/Canada/Spain/South Africa	0	0	0
**IR cotton – global**	0	0	0
**IR soybeans – South America**	0	0	0
***Total***	***5,749.6***	***21,101.1***	***13,980.7***

Applying the same approach to estimating the annual soil carbon sequestration levels for each year between 1996 and 2020 and aggregating the findings suggests that the additional amount of soil carbon sequestered since 1996 has been equivalent to 93,745 million kg of carbon or 344,044 million kg of carbon dioxide that has not been released into the global atmosphere, equivalent to taking about 228 million cars off the road over this period. Readers should, however, note that this estimate is likely to significantly overstate the true soil carbon sequestration benefits from the adoption of RT/NT systems over this 24-year period because some of the additional soil carbon sequestration gains from RT/NT systems will have been lost from some subsequent plowing of land in these crops and production systems.

Estimating these possible losses that may arise from subsequent plowing would be complex and difficult to undertake. One study from the USA (Claassen R et al. 2018^40^) estimated that approximately 20% of the combined area of corn, soybeans, cotton and wheat in the USA were in continuous NT/RT production systems during the period 2012–2016. If this estimate was used as the basis for estimating the cumulative reductions soil carbon associated with GM HT crop adoption-facilitated NT/RT farming in North and South American agriculture, this would equate to a saving of 18,750 million kg of soil carbon and 68,809 million kg of carbon dioxide. In considering these estimates of impact of GM HT technology on soil carbon storage levels and carbon dioxide emission savings, it should be noted that the study findings of Claessen et al., 2018^40^ were US-specific and therefore the finds may not be applicable for estimating impacts in South American countries, especially as the most recent data relating to the share of NT/RT in GM HT soybean production in South America continue to show very high levels of retention of NT/RT (90% plus) compared to some fall back having taken place in the USA.

Overall, it is not possible to confidently estimate cumulative soil sequestration gains that take into account reversions to conventional tillage because of a lack of data.

Returning to the 2020 analysis of carbon emission savings from both sources of fuel-related savings and soil carbon storage, aggregating these benefits results in the total carbon dioxide savings in 2020 being equal to about 23,631 million kg, equivalent to taking 15.6 million cars off the road for a year. This is equal to 49% of registered cars in the UK.

## Conclusions

GM crop technology has been used around the world for nearly 25 years. The technology has helped farmers adapt their weed and pest control practices and become more efficient with their use of crop protection products. In turn, this has contributed to changing agriculture’s carbon footprint by reducing the amount of fuel used to apply crop protection products. It has also helped many farmers in North and South America to adopt (and remain in) more sustainable practices such as reduced and no tillage. This has decreased the use of fossil fuels for plowing and facilitated more carbon to be retained in the soil. This has led to a decrease in carbon emissions from cropping agriculture which are permanent in the case of reduced fuel use.

In relation to GM HT crops, however, over reliance on the use of glyphosate by farmers, in many regions, has contributed to the development of weed resistance. As a result, farmers have, over the last 20 years, adopted more integrated weed management strategies incorporating a mix of herbicides and non-herbicide-based weed control practices. This means that the magnitude of carbon emission savings each year associated with the facilitating role of GM HT crops in the adoption of NT and RT systems is likely to have decreased as some farmers have reverted to making use of plowing as part of weed control practices of herbicide tolerant weeds. This also suggests that some of the cumulative soil sequestration benefits associated with farmers remaining in permanent NT/RT production systems will have been lost. It is, however, not possible to provide reasonable estimates of the possible cumulative levels of soil carbon sequestration due to a lack of available and relevant data.

Nevertheless, despite these developments, the adoption of GM HT crop technology in 2020 continues to deliver lower levels of carbon dioxide emissions relative to the conventional alternative. The carbon dioxide savings associated with the facilitating role of GM HT crop technology in this study are also consistent with the findings of other studies such as Sutherland C et al., 2021^41^ and Rodriguez A et al., 2021.^42^

## APPENDIX 1: CARBON SAVING ESTIMATES

**Table:** US soybeans: permanent reduction in tractor fuel consumption and reduction in carbon dioxide emissions (1996–2020).

	Annual reduction based on 1996 average (liters/ha)	Crop area (million ha)	Total fuel saving (million liters)	Carbon dioxide (million kg)
1996	0.00	25.98	0.00	0.00
1997	0.41	28.33	11.60	30.98
1998	0.80	29.15	23.38	62.41
1999	0.92	29.84	27.38	73.10
2000	1.41	30.15	42.58	113.69
2001	2.40	29.99	72.01	192.26
2002	2.68	29.54	79.10	211.19
2003	2.95	29.71	87.49	233.61
2004	2.89	30.28	87.52	233.68
2005	2.58	28.88	74.55	199.04
2006	1.74	30.56	53.19	142.01
2007	3.91	25.75	100.79	269.10
2008	1.29	30.21	38.87	103.77
2009	1.79	30.91	55.29	147.62
2010	3.22	31.56	101.75	271.67
2011	3.22	30.05	96.88	258.68
2012	2.90	30.82	89.43	238.78
2013	5.79	30.70	177.66	474.35
2014	2.92	33.42	97.52	260.37
2015	3.36	33.12	111.44	297.53
2016	1.87	33.48	62.46	166.78
2017	2.33	36.24	84.49	225.59
2018	1.10	35.66	39.23	104.75
2019	1.20	30.33	36.30	96.92
2020	1.07	33.31	35.66	95.21
**Total**			**1,686.55**	**4,503.10**

Assumption: baseline fuel usage is the 1996 level of 36.6 liters/ha.

## Appendix

**Table:** US soybean: potential additional soil carbon sequestration (1996 to 2020).

	Annual increase in carbon sequestered based on 1996 average (kg carbon/ha)	Crop area (million ha)	Total additional carbon sequestered (million kg)	Total additional Carbon dioxide sequestered (million kg)
1996	0.0	26.0	0.00	0.00
1997	1.4	28.3	38.34	141
1998	2.8	29.1	80.93	297
1999	3.3	29.8	99.20	364
2000	5.2	30.1	156.72	575
2001	8.9	30.0	265.69	975
2002	10.0	29.5	296.63	1,089
2003	11.1	29.7	328.58	1,206
2004	10.9	30.3	328.68	1,206
2005	9.0	28.9	259.54	952
2006	5.3	30.6	162.98	598
2007	14.1	25.8	362.00	1,329
2008	3.9	30.2	118.43	435
2009	5.8	30.9	178.52	655
2010	11.5	31.6	363.72	1,335
2011	11.5	30.1	346.34	1,271
2012	10.7	30.8	328.84	1,207
2013	21.6	30.7	662.98	2,433
2014	10.4	33.4	346.53	1,272
2015	12.2	33.1	405.15	1,487
2016	6.2	33.5	206.14	757
2017	8.0	36.2	291.08	1,068
2018	3.5	35.7	126.21	463
2019	3.7	30.3	113.56	417
2020	3.3	33.3	110.90	407
**Total**			**5,977.67**	**21,938.05**

Assumption: carbon sequestration remains at the 1996 level of −102.9 kg carbon/ha/year.

## Appendix

**Table:** Argentine soybean: permanent reduction in tractor fuel consumption and reduction in carbon dioxide emissions (1996–2020).

	Annual reduction based on 1996 average of 39.1 (liters/ha)	Crop area (million ha)	Total fuel saving (million liters)	Carbon dioxide (million kg)
1996	0.0	5.9	0.0	0.00
1997	2.3	6.4	14.7	39
1998	3.1	7.0	21.5	57
1999	2.7	8.2	21.9	59
2000	3.0	10.6	31.6	84
2001	5.8	11.5	67.2	179
2002	8.3	13.0	107.3	287
2003	9.8	13.5	132.2	353
2004	11.7	14.3	167.4	447
2005	10.7	15.2	163.0	435
2006	11.0	16.2	177.4	474
2007	12.3	16.6	204.2	545
2008	13.7	16.8	230.4	615
2009	13.2	18.6	245.9	657
2010	13.7	18.2	249.8	667
2011	14.3	18.6	265.5	709
2012	14.3	19.4	276.3	738
2013	15.1	19.8	298.0	796
2014	14.3	19.8	282.4	754
2015	14.3	19.4	277.0	739
2016	14.0	18.6	260.5	696
2017	13.7	16.3	224.1	598
2018	13.5	16.6	223.2	596
2019	14.5	16.7	243.3	649
2020	15.1	16.5	248.5	663
**Total**			**4,433.24**	**11,837**

Note: Based on 21.89 liters/ha for NT and 49.01 liters/ha for CT.

## Appendix

**Table:** Argentine soybean: potential additional soil carbon sequestration (1996 to 2020).

	Annual increase in carbon sequestered based on 1996 average (kg carbon/ha)	Crop area (million ha)	Total additional carbon sequestered (million kg)	Total additional Carbon dioxide sequestered (million kg)
1996	-	5.91	-	-
1997	16.92	6.39	108.17	397
1998	22.80	6.95	158.52	582
1999	19.77	8.18	161.68	593
2000	22.03	10.59	233.27	856
2001	43.09	11.50	495.53	1,819
2002	61.05	12.96	791.51	2,905
2003	72.20	13.50	974.71	3,577
2004	86.07	14.34	1,234.69	4,531
2005	79.08	15.20	1,202.00	4,411
2006	81.02	16.15	1,308.48	4,802
2007	90.79	16.59	1,505.72	5,526
2008	101.33	16.77	1,699.00	6,235
2009	97.49	18.60	1,813.37	6,655
2010	101.23	18.20	1,842.45	6,762
2011	105.28	18.60	1,958.28	7,187
2012	105.28	19.35	2,037.25	7,477
2013	111.28	19.75	2,197.86	8,066
2014	105.28	19.78	2,082.52	7,643
2015	105.28	19.40	2,042.51	7,496
2016	103.28	18.60	1,921.08	7,050
2017	101.28	16.32	1,652.76	6,066
2018	99.28	16.58	1,645.72	6,040
2019	107.28	16.72	1,793.94	6,584
2020	111.28	16.47	1,832.48	6,725
**Total**			**32,693.50**	**119,985**

Assumption: NT = +175 kg carbon/ha/yr, Conventional Tillage CT = −25 kg carbon/ha/yr.

## Appendix

**Table:** Brazil (3 southernmost states) soybean: permanent reduction in tractor fuel consumption and reduction in carbon dioxide emissions (1997–2020).

	Annual reduction based on 1997 average of 40.9 (liters/ha)	Crop area (million ha)	Total fuel saving (million liters)	Carbon dioxide (million kg)
1997	0.00	6.19	0.00	0.00
1998	1.36	6.12	8.30	22.15
1999	2.71	6.05	16.40	43.80
2000	4.07	5.98	24.34	65.00
2001	5.42	6.84	37.09	99.03
2002	6.78	7.49	50.76	135.53
2003	8.14	8.21	66.83	178.43
2004	9.49	8.59	81.52	217.65
2005	10.85	8.30	89.98	240.26
2006	12.20	8.25	100.65	268.73
2007	12.20	8.19	99.89	266.71
2008	13.56	8.23	111.56	297.86
2009	14.37	8.90	127.94	341.60
2010	14.92	9.13	136.24	363.75
2011	14.92	9.11	135.83	362.66
2012	15.46	9.88	152.79	407.95
2013	16.27	10.49	170.74	455.87
2014	16.27	11.07	180.20	481.13
2015	16.27	11.55	187.87	501.60
2016	16.27	11.46	186.47	497.88
2017	16.27	11.84	192.58	514.19
2018	16.27	11.88	193.30	516.12
2019	16.27	12.09	196.65	525.05
2020	16.27	12.38	201.37	537.66
**Total**			**2,749.29**	**7,340.60**

Note: Based on 21.89 liters/ha for NT and RT and 49.01 liters/ha for CT.

## Appendix

**Table:** Brazil (3 southernmost states) soybean: potential additional soil carbon sequestration (1997 to 2020).

	Annual increase in carbon sequestered based on 1997 average (kg carbon/ha)	Crop area (million ha)	Total addition carbon sequestered (million kg)	Total addition Carbon dioxide sequestered (million kg)
1997	0.0	6.2	0.00	0.00
1998	10.0	6.1	61.19	224.57
1999	20.0	6.0	120.98	444.00
2000	30.0	6.0	179.52	658.84
2001	40.0	6.8	273.52	1,003.82
2002	50.0	7.5	374.35	1,373.86
2003	60.0	8.2	492.84	1,808.72
2004	70.0	8.6	601.16	2,206.26
2005	80.0	8.3	663.60	2,435.41
2006	90.0	8.2	742.23	2,723.98
2007	90.0	8.2	736.65	2,703.51
2008	100.0	8.2	822.70	3,019.31
2009	106.0	8.9	943.51	3,462.67
2010	110.0	9.1	1,004.69	3,687.19
2011	110.0	9.1	1,001.67	3,676.13
2012	114.0	9.9	1,126.76	4,135.23
2013	120.0	10.5	1,259.12	4,620.99
2014	120.0	11.1	1,328.89	4,877.03
2015	120.0	11.5	1,385.45	5,084.59
2016	120.0	11.5	1,375.15	5,046.81
2017	120.0	11.8	1,420.21	5,212.18
2018	120.0	11.9	1,425.55	5,231.78
2019	120.0	12.1	1,450.21	5,322.28
2020	120.0	12.4	1,485.04	5,450.08
**Total**			**20,274.99**	**74,409.23**

Assumption: NT/RT = +175 kg carbon/ha/yr, CT = −25 kg carbon/ha/yr.

## Appendix

**Table:** Canada soybeans: permanent reduction in tractor fuel consumption and reduction in carbon dioxide emissions (1997–2020).

	Annual reduction based on 1996 average (liters/ha)	Crop area (million ha)	Total fuel saving (million liters)	Carbon dioxide (million kg)
1997	0.00	1.06	0.00	0.00
1998	1.15	0.98	1.13	3.00
1999	2.19	1.00	2.20	5.88
2000	2.40	1.06	2.55	6.80
2001	2.71	1.07	2.90	7.74
2002	3.86	1.02	3.95	10.55
2003	5.01	1.04	5.23	13.96
2004	6.26	1.20	7.54	20.14
2005	6.56	1.18	7.72	20.61
2006	6.87	1.21	8.34	22.26
2007	7.41	1.18	8.74	23.34
2008	7.58	1.20	9.11	24.31
2009	7.74	1.38	10.70	28.57
2010	7.91	1.48	11.68	31.20
2011	8.08	1.56	12.59	33.62
2012	7.97	1.68	13.38	35.73
2013	8.14	1.87	15.22	40.63
2014	8.04	2.24	17.96	47.96
2015	8.04	2.19	17.56	46.89
2016	8.04	2.21	17.72	47.32
2017	8.04	2.94	23.59	62.98
2018	8.04	2.54	20.41	54.51
2019	8.04	2.27	18.25	48.74
2020	8.04	2.04	16.40	43.80
**Total**			***254.88***	***680.54***

Assumption: baseline fuel usage is the 1996 level of 40.4 liters/ha.

## Appendix

**Table:** Canada soybean: potential additional soil carbon sequestration (1997 to 2020).

	Annual increase in carbon sequestered based on 1996 average (kg carbon/ha)	Crop area (million ha)	Total additional carbon sequestered (million kg)	Total additional Carbon dioxide sequestered (million kg)
1997	0.0	1.1	0.00	0.00
1998	4.4	1.0	4.31	15.83
1999	8.5	1.0	8.53	31.32
2000	9.1	1.1	9.65	35.42
2001	10.0	1.1	10.69	39.23
2002	14.4	1.0	14.74	54.11
2003	18.8	1.0	19.63	72.05
2004	23.5	1.2	28.31	103.90
2005	24.7	1.2	29.05	106.60
2006	25.9	1.2	31.44	115.39
2007	27.9	1.2	32.92	120.82
2008	28.6	1.2	34.38	126.16
2009	29.3	1.4	40.49	148.61
2010	30.0	1.5	44.31	162.62
2011	30.7	1.6	47.86	175.65
2012	30.4	1.7	51.01	187.21
2013	31.1	1.9	58.13	213.32
2014	30.8	2.2	68.84	252.64
2015	30.8	2.2	67.30	246.98
2016	30.8	2.2	67.91	249.24
2017	30.8	2.9	90.40	331.76
2018	30.8	2.5	78.23	287.11
2019	30.8	2.3	69.95	256.70
2020	30.8	2.0	62.86	230.71
**Total**			**970.95**	**3,563.40**

Assumption: carbon sequestration remains at the 1996 level of −115.7 kg carbon/ha/year.

## Appendix

**Table:** Bolivia, Paraguay and Uruguay soybeans: permanent reduction in tractor fuel consumption and reduction in carbon dioxide emissions (1999–2020).

	Annual reduction based on 1996 average (liters/ha)	Crop area (million ha)	Total fuel saving (million liters)	Carbon dioxide (million kg)
1999	0.0	1.8	0.0	0.00
2000	1.4	1.8	2.4	6.53
2001	2.7	2.0	5.4	14.32
2002	4.1	2.1	8.6	22.93
2003	5.4	2.2	12.1	32.40
2004	6.8	3.0	20.2	53.81
2005	8.1	3.3	26.7	71.26
2006	9.5	3.4	31.9	85.23
2007	9.5	3.8	36.4	97.32
2008	9.5	4.0	37.9	101.25
2009	9.5	4.4	41.7	111.26
2010	10.0	4.8	47.7	127.45
2011	11.2	4.6	51.8	138.34
2012	11.2	5.1	57.1	152.58
2013	11.2	5.7	63.3	168.97
2014	11.2	6.0	67.3	179.82
2015	11.2	5.8	64.7	172.85
2016	11.2	5.5	62.0	165.47
2017	11.2	5.9	66.2	176.71
2018	11.2	5.7	63.6	169.85
2019	11.8	5.6	65.7	175.38
2020	11.8	5.6	66.5	177.65
**Total**			**899.39**	**2,401.38**

Note: Based on 21.89 liters/ha for NT and RT and 49.01 liters/ha for CT.

## Appendix

**Table:** Bolivia, Paraguay and Uruguay soybean: potential additional soil carbon sequestration (1999 to 2020).

	Annual increase in carbon sequestered based on 1996 average (kg carbon/ha)	Crop area (million ha)	Total additional carbon sequestered (million kg)	Total additional Carbon dioxide sequestered (million kg)
1999	0.0	1.8	0.0	0.00
2000	10.0	1.8	18.0	66.15
2001	20.0	2.0	39.5	145.13
2002	30.0	2.1	63.3	232.46
2003	40.0	2.2	89.5	328.41
2004	50.0	3.0	148.6	545.47
2005	60.0	3.3	196.8	722.37
2006	70.0	3.4	235.4	863.92
2007	70.0	3.8	268.8	986.50
2008	70.0	4.0	279.7	1,026.32
2009	70.0	4.4	307.3	1,127.79
2010	74.0	4.8	352.0	1,291.91
2011	82.6	4.6	382.1	1,402.33
2012	82.6	5.1	421.4	1,546.63
2013	82.6	5.7	466.7	1,712.75
2014	82.6	6.0	496.7	1,822.79
2015	82.6	5.8	477.4	1,752.16
2016	82.6	5.5	457.0	1,677.28
2017	82.6	5.9	488.1	1,791.27
2018	82.8	5.7	469.1	1,721.76
2019	87.0	5.6	484.4	1,777.75
2020	87.0	5.6	490.7	1,800.80
**Total**			**6,632.68**	**24,341.94**

Assumption: NT = +175 kg carbon/ha/yr, Conventional Tillage CT = −25 kg carbon/ha/yr.

## Appendix

**Table:** US maize: permanent reduction in tractor fuel consumption and reduction in carbon dioxide emissions (1998–2020).

	Annual reduction based on 1997 average (liters/ha)	Crop area (million ha)	Total fuel saving (million liters)	Carbon dioxide (million kg)
1997	0.00	32.19	0.00	0.00
1998	−0.55	32.44	−17.83	−47.60
1999	−0.92	31.32	−28.74	−76.73
2000	−1.29	32.19	−41.39	−110.51
2001	−1.29	30.64	−39.43	−105.27
2002	−1.29	31.93	−41.13	−109.82
2003	−1.08	31.81	−34.32	−91.65
2004	−0.87	32.47	−28.24	−75.41
2005	3.53	33.10	116.84	311.95
2006	3.42	31.70	108.45	289.57
2007	3.05	37.88	115.65	308.78
2008	4.41	31.82	140.30	374.60
2009	6.52	32.21	210.01	560.73
2010	6.33	32.78	207.64	554.40
2011	3.95	34.35	135.65	362.19
2012	4.13	35.36	145.95	389.68
2013	4.41	35.48	156.62	418.17
2014	6.48	33.64	217.86	581.68
2015	6.48	32.68	211.76	565.39
2016	4.67	35.11	163.87	437.54
2017	3.61	33.48	121.01	323.09
2018	4.34	33.08	143.69	383.65
2019	4.49	32.92	147.82	394.67
2020	4.36	33.37	145.36	388.11
**Total**			**2,257.39**	**6,027.22**

Assumption: baseline fuel usage is the 1997 level of 42.6 liters/ha.

## Appendix

**Table:** US maize: potential additional soil carbon sequestration (1998 to 2020).

	Annual increase in carbon sequestered based on 1997 average (kg carbon/ha)	Crop area (million ha)	Additional carbon sequestered (million kg)	Additional carbon dioxide sequestered (million kg)
1997	0.0	32.2	0.00	0.00
1998	−5.7	32.4	−183.41	−673.13
1999	−9.4	31.3	−294.20	−1,079.72
2000	−13.1	32.2	−422.85	−1,551.87
2001	−13.2	30.6	−403.30	−1,480.12
2002	−13.2	31.9	−421.26	−1,546.04
2003	−11.1	31.8	−351.70	−1,290.73
2004	−8.9	32.5	−289.56	−1,062.68
2005	35.7	33.1	1,182.31	4,339.09
2006	34.6	31.7	1,096.74	4,025.05
2007	30.7	37.9	1,164.52	4,273.78
2008	44.8	31.8	1,425.16	5,230.35
2009	66.7	32.2	2,148.54	7,885.12
2010	64.8	32.8	2,123.58	7,793.55
2011	40.0	34.4	1,374.40	5,044.06
2012	42.0	35.4	1,485.39	5,451.39
2013	44.8	35.5	1,591.05	5,839.16
2014	66.2	33.6	2,228.31	8,177.91
2015	66.3	32.7	2,166.55	7,951.23
2016	47.5	35.1	1,666.96	6,117.73
2017	36.6	33.5	1,225.70	4,498.33
2018	44.1	33.1	1,460.15	5,358.74
2019	45.4	32.9	1,493.05	5,479.51
2020	44.4	33.4	1,481.63	5,437.57
**Total**			**22,947.76**	**84,218.29**

Assumption: carbon sequestration remains at the 1997 level of 122.5 kg carbon/ha/year.

## Appendix

**Table:** Canada maize: permanent reduction in tractor fuel consumption and reduction in carbon dioxide emissions (1999–2020).

	Annual reduction based on 1996 average (liters/ha)	Crop area (million ha)	Total fuel saving (million liters)	Carbon dioxide (million kg)
1999	0.00	1.15	0.00	0.00
2000	0.15	1.09	0.16	0.44
2001	0.38	1.27	0.48	1.27
2002	1.45	1.28	1.86	4.95
2003	2.52	1.14	2.86	7.63
2004	3.66	1.46	5.34	14.26
2005	3.95	1.30	5.15	13.75
2006	4.24	1.33	5.65	15.08
2007	4.73	1.60	7.56	20.19
2008	4.90	1.20	5.90	15.75
2009	5.07	1.14	5.79	15.46
2010	5.24	1.20	6.30	16.83
2011	5.41	1.20	6.50	17.35
2012	5.33	1.42	7.56	20.19
2013	5.50	1.48	8.14	21.74
2014	5.43	1.23	6.66	17.78
2015	5.43	1.31	7.12	19.01
2016	5.43	1.33	7.19	19.19
2017	5.43	1.41	7.63	20.37
2018	5.43	1.43	7.76	20.73
2019	5.43	1.45	7.87	21.02
2020	5.43	1.40	7.61	20.31
**Total**			***121.08***	***323.29***

Assumption: baseline fuel usage is the 1999 level of 45.2 liters/ha.

## Appendix

**Table:** Canada maize: potential additional soil carbon sequestration (1999 to 2020).

	Annual increase in carbon sequestered based on 1996 average (kg carbon/ha)	Crop area (million ha)	Total additional carbon sequestered (million kg)	Total additional Carbon dioxide sequestered (million kg)
1999	0.0	1.1	0.00	0.00
2000	−2.8	1.1	−3.04	−11.15
2001	−2.3	1.3	−2.90	−10.66
2002	4.3	1.3	5.49	20.16
2003	16.8	1.1	19.08	70.02
2004	27.7	1.5	40.35	148.09
2005	14.9	1.3	19.38	71.13
2006	17.4	1.3	23.16	85.01
2007	18.4	1.6	29.40	107.90
2008	15.7	1.2	18.93	69.49
2009	9.8	1.1	11.23	41.21
2010	12.5	1.2	15.08	55.34
2011	11.9	1.2	14.27	52.36
2012	11.1	1.4	15.79	57.93
2013	11.9	1.5	17.66	64.83
2014	11.1	1.2	13.68	50.20
2015	11.1	1.3	14.63	53.68
2016	11.1	1.3	14.77	54.21
2017	11.1	1.4	15.67	57.52
2018	11.1	1.4	15.95	58.54
2019	11.1	1.5	16.18	59.36
2020	11.1	1.4	15.63	57.36
**Total**			**330.39**	**1,212.53**

Assumption: carbon sequestration remains at the 1999 level of 90.7 kg carbon/ha/year.

## Appendix

**Table:** Canadian canola: permanent reduction in tractor fuel consumption and reduction in carbon dioxide emissions (1996–2020).

	Annual reduction based on 1996 average 30.6 (l/ha)	Crop area (million ha)	Total fuel saving (million liters)	Carbon dioxide (million kg)
1996	0.0	3.5	0.0	0.00
1997	0.9	4.9	4.3	11.51
1998	0.9	5.4	4.8	12.83
1999	0.9	5.6	4.9	13.15
2000	0.9	4.9	4.3	11.48
2001	1.8	3.8	6.7	17.89
2002	2.7	3.3	8.7	23.12
2003	3.5	4.7	16.6	44.32
2004	4.4	4.9	21.9	58.35
2005	5.3	5.5	29.2	77.85
2006	6.2	5.2	32.5	86.64
2007	6.5	5.9	38.7	103.36
2008	7.1	6.5	46.0	122.77
2009	8.0	6.4	50.8	135.59
2010	8.8	6.5	57.7	153.93
2011	8.9	7.5	66.1	176.54
2012	8.9	8.6	76.0	202.86
2013	8.9	7.8	69.1	184.61
2014	8.9	8.3	73.8	197.16
2015	8.9	8.1	71.5	191.00
2016	8.9	8.1	71.9	191.85
2017	8.9	9.3	82.1	219.12
2018	8.9	9.1	80.7	215.50
2019	8.9	8.5	74.8	199.81
2020	8.9	8.3	73.6	196.60
**Total**			**1,066.6**	**2,847.8**

Note: Fuel usage NT/RT = 17.3 liters/ha CT = 35 liters/ha.

## Appendix

**Table:** Canadian canola: potential additional soil carbon sequestration (1996 to 2020).

	Annual increase in carbon sequestered based on 1996 average (kg carbon/ha)	Crop area (million ha)	Total carbon sequestered (million kg)	Carbon dioxide (million kg)
1997	0.0	3.5	0.00	-
1998	3.3	4.9	15.83	58.09
1999	3.3	5.4	17.64	64.75
2000	3.3	5.6	18.08	66.37
2001	3.3	4.9	15.79	57.96
2002	6.5	3.8	24.60	90.30
2003	9.8	3.3	31.80	116.71
2004	13.0	4.7	60.96	223.72
2005	16.3	4.9	80.26	294.55
2006	19.5	5.5	107.07	392.96
2007	22.8	5.2	119.17	437.36
2008	24.1	5.9	142.16	521.72
2009	26.0	6.5	168.86	619.71
2010	29.3	6.4	186.50	684.44
2011	32.5	6.5	211.72	777.00
2012	32.5	7.5	242.81	891.10
2013	32.5	8.6	279.01	1,023.98
2014	32.5	7.8	253.91	931.84
2015	32.5	8.3	271.18	995.23
2016	32.5	8.1	262.70	964.10
2017	32.5	8.1	263.87	968.39
2018	32.5	9.3	301.37	1,106.04
2019	32.5	9.1	296.40	1,087.79
2020	32.5	8.5	274.82	1,008.59
2020	32.5	8.3	270.40	992.37
**Total**			**3,916.91**	**14,375.06**

Note: NT/RT = +55 kg of carbon/ha/yr CT = −10 kg of carbon/ha/yr.

## Appendix

**Table:** Permanent reduction in global tractor fuel consumption and carbon dioxide emissions resulting from the cultivation of GM IR maize in Brazil (2008–2020).

	Total corn area Brazil (million ha)	Insect resistant area (million ha)	Total spray runs saved (million ha)	Fuel saving (million liters)	CO2 emissions saved (million kg)
2008	13.44	1.45	4.35	3.65	9.76
2009	12.99	4.76	14.28	12.00	32.03
2010	13.81	7.44	22.32	18.75	50.06
2011	15.12	8.68	26.04	21.88	58.41
2012	15.82	10.95	32.85	27.59	73.67
2013	15.27	11.88	35.64	29.94	79.93
2014	15.82	11.91	35.73	30.01	80.14
2015	15.75	12.38	37.15	31.21	83.32
2016	17.59	14.88	44.64	37.50	100.13
2017	16.60	13.68	41.04	34.47	92.03
2018	17.20	13.95	41.85	35.15	93.86
2019	18.53	16.25	48.75	40.95	109.33
2020	19.83	18.05	54.14	45.47	121.42
**Total**			**438.77**	**368.56**	**984.07**

## Appendix

**Table:** Permanent reduction in global tractor fuel consumption and carbon dioxide emissions resulting from the cultivation of GM IR maize in the USA, Canada, Spain and South Africa (1996–2020).

	Number of applications saved (‘000s)	Fuel saving from less spray runs liters (‘000s)	CO2 emissions saved kgs (‘000s)
1996	301	253	675
1997	2,509	2,108	5,627
1998	3,378	2,838	7,577
1999	3,272	2,748	7,338
2000	3,431	2,882	7,696
2001	3,331	2,798	7,471
2002	3,512	2,950	7,877
2003	3,557	2,988	7,978
2004	3,847	3,231	8,627
2005	3,875	3,257	8,690
2006	4,327	3,635	9,705
2007	5,218	4,383	11,703
2008	4,691	3,941	10,522
2009	5,137	4,315	11,522
2010	5,074	4,262	11,380
2011	5,173	4,345	11,602
2012	5,484	4,607	12,300
2013	5,508	4,626	12,353
2014	5,296	4,449	11,879
2015	4,893	4,110	10,973
2016	5,447	4,575	12,216
2017	5,257	4,415	11,789
2018	5,208	4,375	11,681
2019	5,234	4,396	11,738
2020	5,280	4,435	11,842
**Total**	**108,241**	**90,922**	**242,762**

Assumptions:

^1^Number of applications saved (based on one per ha of lowest of total GM IR maize area or area pre-GM IR maize that was traditionally sprayed for treatment of pests targeted by GM IR technology.

^2^Fuel saving per ha 0.84 liters/ha.

## Appendix

**Table:** Permanent reduction in global tractor fuel consumption and carbon dioxide emissions resulting from the cultivation of GM IR cotton (1996–2020).

	Total cotton area in GM IR growing countries excluding Burkina Faso, India, Pakistan, Myanmar, Sudan and China (million ha)	GM IR area excluding Burkina Faso, India, Pakistan, Myanmar, Sudan and China (million ha)	Total spray runs saved (million ha)	Fuel saving (million liters)	CO2 emissions saved (million kg)
1996	6.64	0.86	3.45	2.90	7.73
1997	6.35	0.92	3.67	3.09	8.24
1998	7.20	1.05	4.20	3.53	9.43
1999	7.42	2.11	8.44	7.09	18.92
2000	7.29	2.43	9.72	8.17	21.81
2001	7.25	2.55	10.18	8.55	22.84
2002	6.36	2.17	8.69	7.30	19.49
2003	5.34	2.17	8.69	7.30	19.49
2004	6.03	2.79	11.17	9.38	25.05
2005	6.34	3.21	12.84	10.78	28.79
2006	7.93	3.95	15.79	13.26	35.40
2007	6.08	3.25	12.99	10.91	29.13
2008	4.51	2.53	10.11	8.50	22.68
2009	5.33	2.96	11.83	9.94	26.54
2010	7.13	4.59	18.37	15.43	41.21
2011	6.61	4.43	17.70	14.87	39.71
2012	5.71	4.07	16.29	13.68	36.53
2013	5.29	3.75	15.01	12.61	33.66
2014	5.58	4.20	16.80	14.11	37.67
2015	5.00	3.94	15.77	13.25	35.37
2016	5.74	4.64	18.54	15.58	41.59
2017	6.68	5.49	21.96	18.45	49.25
2018	6.63	5.50	22.02	18.49	49.38
2019	7.02	6.18	24.73	20.77	55.46
2020	5.87	5.03	20.12	16.90	45.13
**Total**			**339.08**	**284.83**	**760.50**

Notes: assumptions: 4 applications per ha, 0.84 liters/ha of fuel per insecticide application. Fuel saving per ha 0.84 liters/ha.

## Appendix

**Table:** Permanent reduction in global tractor fuel consumption and carbon dioxide emissions resulting from the cultivation of GM IR soybeans in South America (2013–2020).

	Total corn area Brazil (million ha)	Insect resistant area (million ha)	Total spray runs saved (million ha)	Fuel saving (million liters)	CO2 emissions saved (million kg)
2013		2.11	5.7	4.77	12.74
2014		8.0	26.4	22.17	59.20
2015		17.2	56.7	47.61	127.12
2016		22.3	75.7	63.56	169.72
2017		22.7	78.7	66.13	176.55
2018		25.7	91.2	76.60	204.53
2019		28.5	98.6	82.81	221.09
2020		29.5	101.8	85.55	228.43
**Total**				**449.21**	**1,199.38**

Notes and assumptions:

^1^Countries: Brazil, Argentina, Paraguay and Uruguay.

^2^Number of insecticide applications saved per ha; Brazil 4, Paraguay 2, Argentina and Uruguay 1 each.

^3^Number of applications saved (based on one per ha of lowest of total GM IR soybean area or area pre-GM IR soybeans that was traditionally sprayed for treatment of pests targeted by GM IR technology.

^4^Fuel saving per ha 0.84 liters/ha.
